# Supplementary material for: Precise Filtration of Chronic Myeloid Leukemia Cells by an Ultrathin Microporous Membrane with Backflushing to Minimize Fouling
Source: Membranes (Basel). 2023 Jul 29;13(8):707. doi: 10.3390/membranes13080707 (PMC10456395; doi:10.3390/membranes13080707)
Supplement: Supplementary file 1 [file membranes-13-00707-s001.zip › membranes-2527907-supplementary.pdf]

Supporting Information

Precise Filtration of Chronic Myeloid Leukemia Cells by an Ultrathin Microporous Membrane with Backflushing to Minimize Fouling

Jaehyuk Lee [1,2], Jeongpyo Hong [2], Jungwon Lee [2], Changgyu Lee [2], Tony Kim [2], Young Jeong [2], Kwanghee Kim [3] and Inhwa Jung [1,*]

[1] Department of Mechanical Engineering, Kyung Hee University, Yongin 17104, Republic of Korea; jh.lee@metapore.com
[2] R&D Center, Metapore Co., Ltd., Advanced Institutes of Convergence Technology 8F, Suwon 16229, Republic of Korea; hongjp05@metapore.com (J.H.); jyl9965@metapore.com (J.L.); cglee@metapore.com (C.L.); tonykim@metapore.com (T.K.); young.j@metapore.com (Y.J.)
[3] National NanoFab Center, 291 Daehak-ro, Yuseong-gu, Daejeon 34141, Republic of Korea; khkim@nnfc.re.kr
* Correspondence: ijung@khu.ac.kr

Detailed structure and operation principle of the PHF and TFF system

In Fig.S1, photos of systems for the three different filtering methods are shown. As shown in Fig S1a, in the case of DEF, filtering was performed only by gravitational force without a pump. In the case of TFF, filtering is performed by standing the filter vertically, whereas in the case of DEF and PHF, filtering is performed by placing the filter horizontally. In Fig.S2, detailed structure and operation principle of the PHF and TFF system are shown. The key is to make the piston of the 50ml syringe reciprocate with the syringe pump, thereby creating positive and negative pressure inside the pumping head. For PHF, a syringe pump (Fusion 100, Chemyx, USA) was used. For TFF, two syringe pumps (PSD/4 Syringe Pump Drive, Hamilton, USA) were used. Whereas a single syringe pump applied to PHF was used to generate periodic positive and negative pressure, the two syringe pumps applied to TFF were used for continuous liquid injection. Specifically, the method of reciprocating motion is shown in Fig. S2a. In Fig. S2b, the detailed structure of the PHF is shown, including the housing and silicone gasket. In Fig.S2c, the structure of the TFF housing and silicone gasket is shown. In particular, in the case of a front silicone gasket, a zigzag-shaped flow path for tangential flow path is formed. In Fig.S2d, the periodic motion of the syringe is shown as a function of time. Here, fouling can be effectively removed by synchronizing the motion of the syringe and the solenoid valve to generate backflush.

(a)
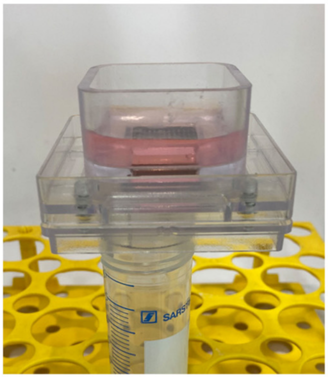
(b)
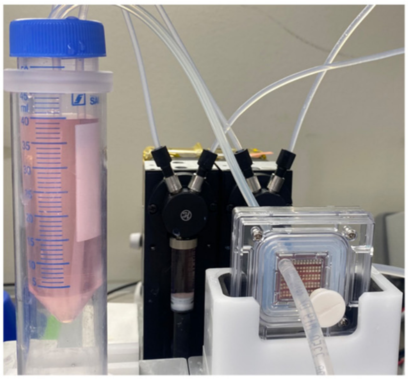
(c)
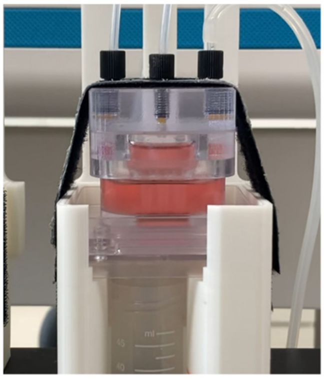

Figure S1. *Photos of DEF, TFF, PHF systems*: (a) a photo of a dead-end filtration system, (b) a photo of a tangential flow filtration system, (c) a photo of a pumping head filtration with backflush system.

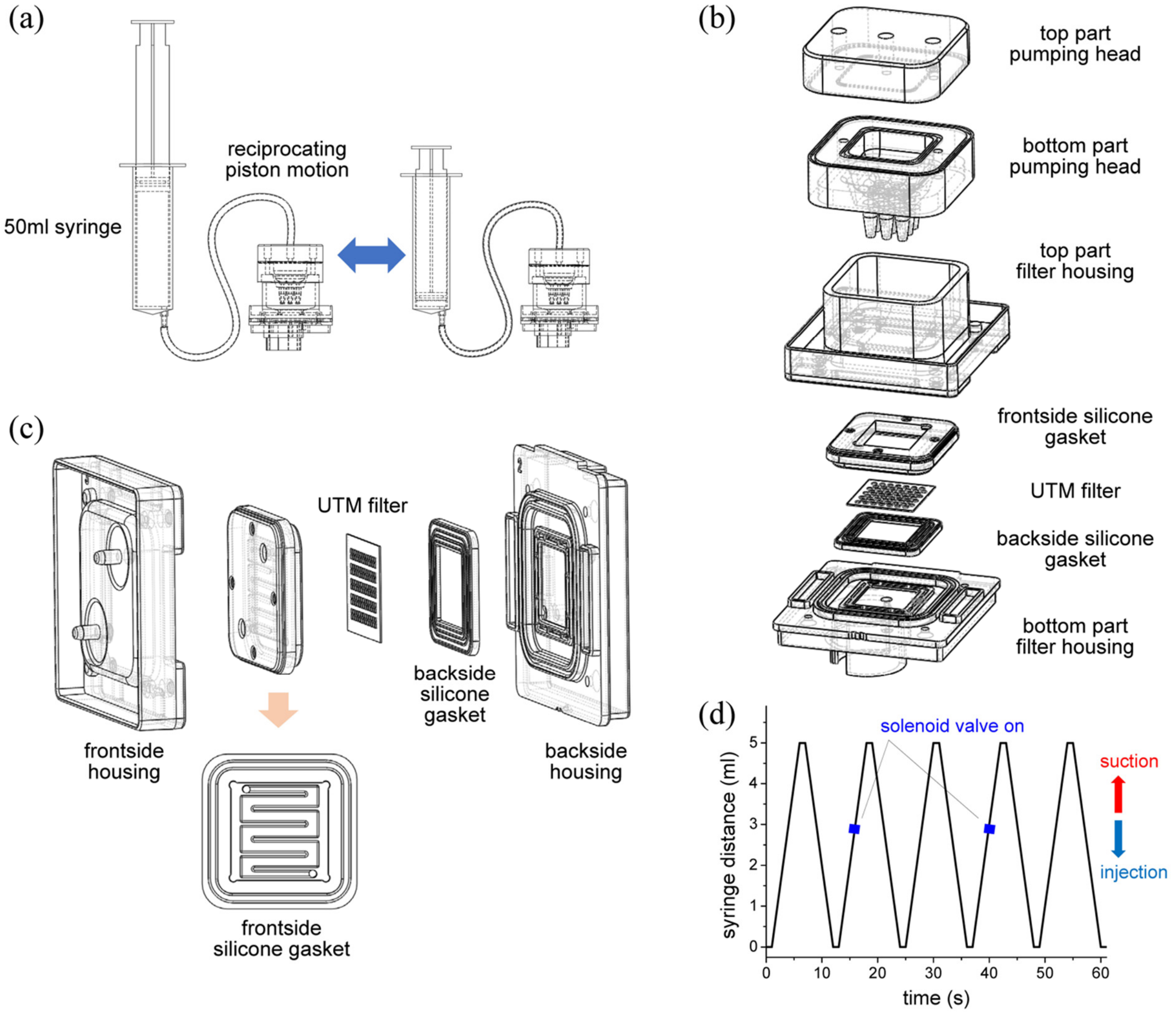

Figure S2. *Details on* the f*iltering system and housing design*: (a) reciprocating motion of the pumping head filtration with backflush (PHF) unit of the filtration system, (b) housing assembly drawing of PHF, (c) housing assembly drawing of tangential flow filtration, (d) syringe distance as a function of time during suction and injection.

Cell recognition and size determination using the artificial intelligence technique

The CSM program is a routine that recognizes cells in the image input by the user and determines the state of these cells through deep learning. The CSM program has been built based on Python. This program can find the shape of a cell using chromaticity, saturation, and brightness, and classify the cell through training. Fig.S3a shows images recognized as cells based on chromaticity, saturation, and brightness. In Fig.S3b, it is the result of converting to a black and white image after recognizing cells in the entire measured image. For cell recognition, a deep learning algorithm was applied. [46, 47] In the deep learning algorithm, a neural network is composed of a 4-step neuron layer and a 2-step dense layer. An optimizer was applied to reduce the loss, and adam and batch size were divided into 32 and 5 classes, respectively. 1000 sheets of data were trained with 200 iterations and showed 98% accuracy. However, since an arbitrary boundary is set in the cell recognition process, the accuracy is

rather limited. Fig.S3c is a picture of the K562 cell before measurement, Fig.S3d is the result of manually measuring, and Fig.S3e is the result of applying the CSM program. Fig.S3f-e is the size distribution of K562 cells generated based on 224 measurement results. Here, Fig.S3f and g show the results of manually measuring and automatically measuring using the CSM program, respectively. It indicates that the result measured manually and the results using the CSM program agrees well.

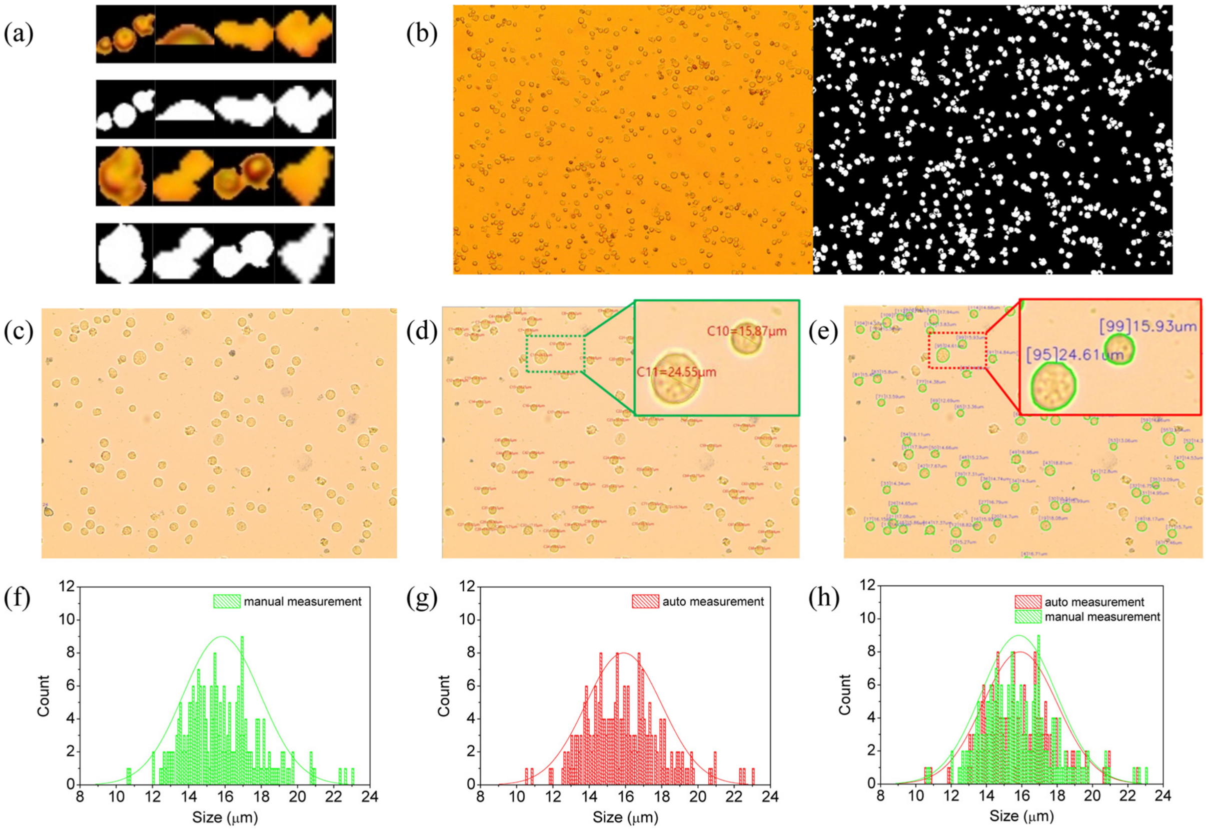

Figure S3. *Operation principles of the cell size measurement (CSM) software*: (a) captured images of various cells,

(b) the original microscope used for capturing cells (left), transformed image after capture (right), (c) the optical microscope image of K562 cells, (d) size determined manually, (e) size determined automatically, (f) manually determined cell size distribution, (g) automatically determined cell size distribution, (h) comparison of manually and automatically determined cell size distributions.

Viscoelastic material modelling of the cell

The viscoelastic properties of the cell material were modeled as a Prony relaxation series. Shear modulus using Prony series parameters is defined as follows [48]:

$$G(t) = G_0\left(1 - \sum_{i=1}^{N} G_i(1 - e^{-\frac{t}{\tau_i}})\right)$$

where G(t) is shear modulus with respect to time, N is the number of series, G_0 is the initial shear modulus, G_i is shear material constant and τ_i is the time constant. To define each parameter, the viscoelasticity of a typical biological tissue was used. [31] Specifically, N was set as 3, G_0 was set as 225.56 Pa, G_1, G_2, G_3 were set as 0.3,

0.35, and 0.1, respectively and τ_1, τ_2, and τ_3 were set as 0.01, 1, and 5, respectively. In Fig.S4, the shear modulus of the cell used for finite element analysis is presented as a function of time.

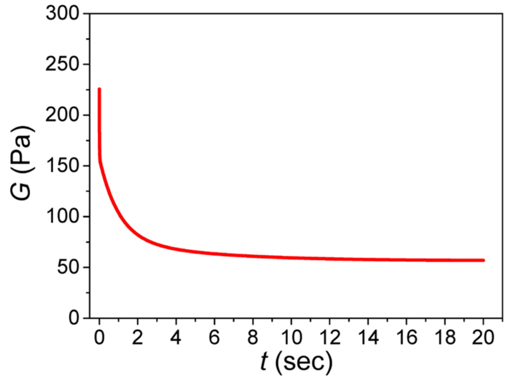

Figure S4. Shear modulus of the cell materials based on Prony series

SEM images and cross-sectional image of UTM filters

In Fig.S5a-e, SEM images of 5~9μm pore diameter filters are shown. In addition, the pore diameter was precisely measured through SEM images. In Fig.S5f, a cross-sectional view of the 5μm membrane is shown. For cross-sectional measurements, the membrane was etched using a focused ion beam (FIB). As a result of precisely measuring the thickness of the membrane through the SEM image, it was measured as 890.3 nm.

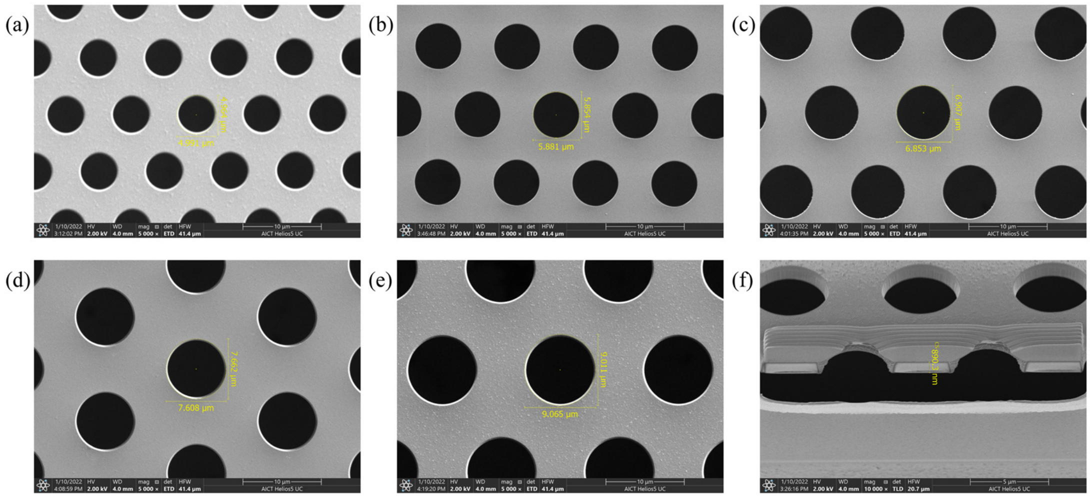

Figure S5. *Pore and membrane structural characteristics*: (a) SEM image of a porous membrane with the pore diameter 5μm, (b) 6μm, (c) 7μm, (d) 8μm, (e) 9μm, (f) cross-sectional view of the 5μm membrane.

Size distribution measurement of a beads mixture

The CSM program presented above is applicable not only to micro-scale cells but also to beads. Applying the developed routine to beads is much more straightforward than to cells. The CSM program can extract the outline of the particle and determine the diameter from the area. Agglomerated particles were set not to be measured. In Fig.S6a, an optical microscope image of a sample mixed with four types of beads is shown. In Fig.S6b, manually measured particle diameters are indicated. In Fig.S6.c, the outlines of the beads recognized through The CSM program are highlighted. In Fig.S6f-e, the size distributions of beads mixture are shown. Here, Fig.S6d and e show

the results of manually measuring and automatically measuring using the CSM program, respectively. It indicates that the result measured manually and the result using the CSM program exhibits a reasonable agreement. Using the CSM program, a large number of particles can be analyzed quickly. In Fig.S7, the results of measuring the distribution of beads before mixing are presented. Notably, as a result of the very precise measurement, it was confirmed that a small amount of beads of a different size than the corresponding particle size was detected.

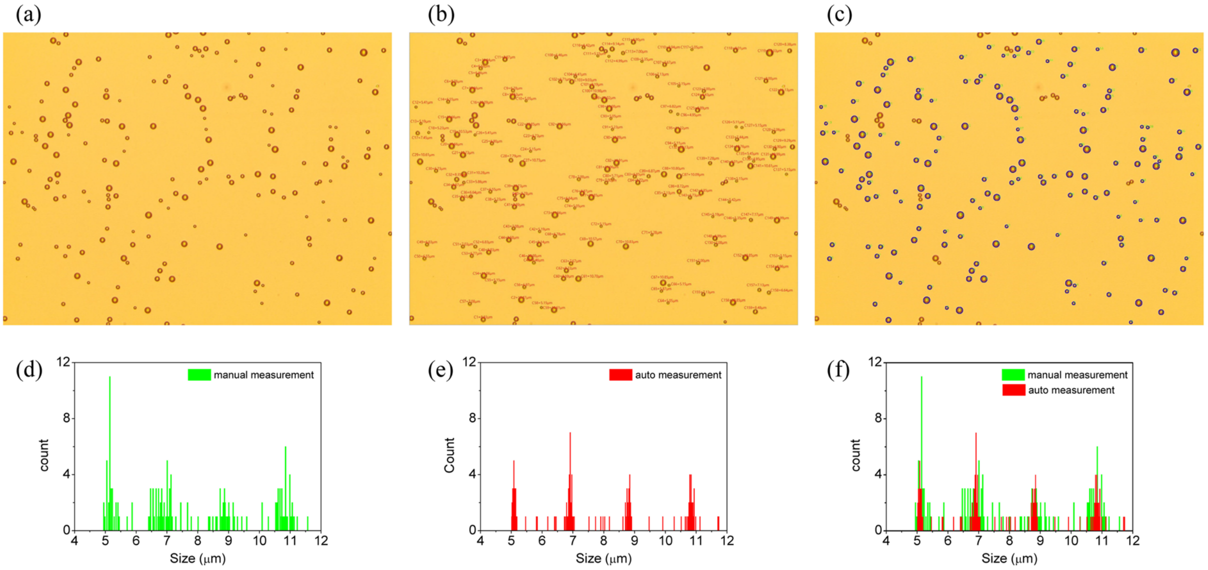

Figure S6. *Size measurement result of beads mixture*: (a) optical microscope images of four sizes of beads, (b) manual measurement, (c) auto measurement, (d) manually measured particle size distribution, (e) automatically measured particle size distribution, (f) relative distribution of manually and automatically measured particle sizes.

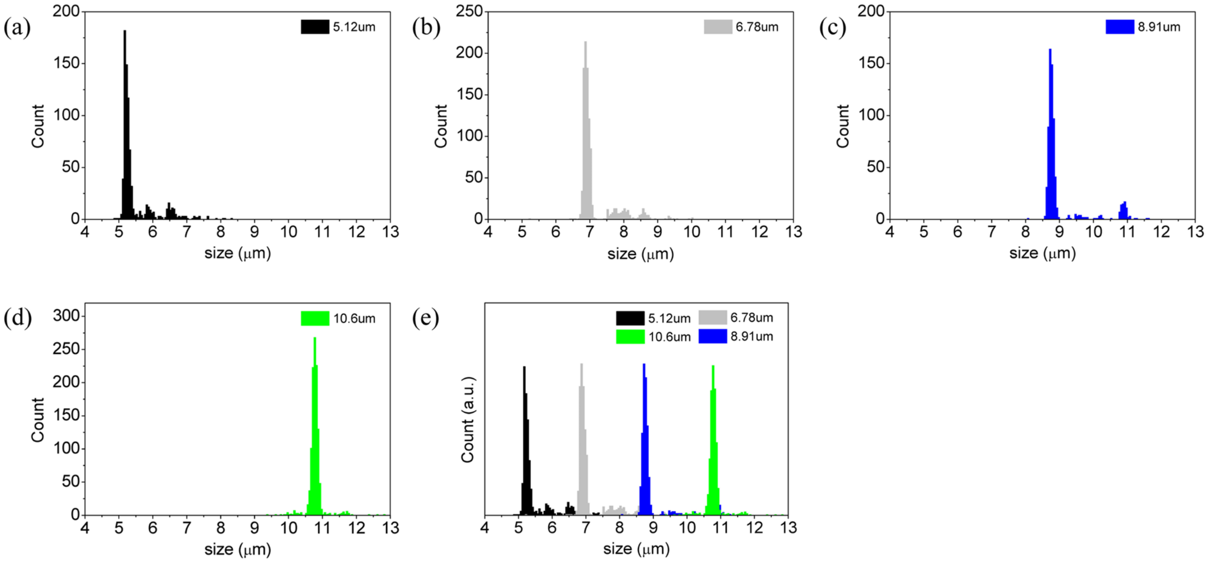

Figure S7. *Size measurement results of individual beads before mixing*: (a) size measurement result of 5.1μm beads,

(b) 6.8μm beads, (c) 8.9μm beads, (d) 10.6μm beads, (e) merged results of all sizes of beads.

Optical microscope image of UTM filters clogged by PS beads

In Fig.S8, the optical microscope image before the UTM-8μm filter is clogged and the image after being clogged by beads during the dead end filtration process are shown. Fig.S8b is a filter image when clogged by beads with

a diameter of 5.1μm, and Fig.S8c shows a filter image when clogged by beads with a diameter of 8.9μm. Since the beads of 5.1μm are smaller than the filter pore diameter, it is judged that they should all pass through the filter, but in reality, a clogging phenomenon occurs due to the formation of a cake layer. In the case of 8.9 μm beads, it can be confirmed that a cake layer was formed because the beads could not pass through the filter.

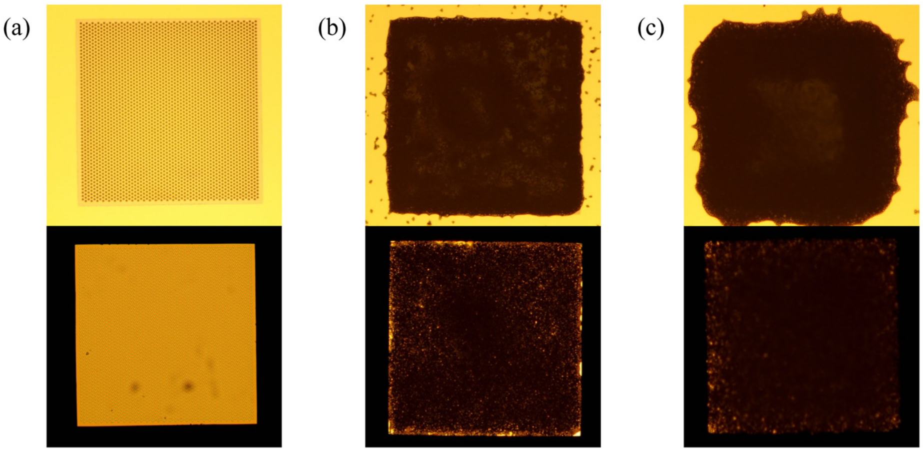

Figure S8. *Optical microscope images of UTM-8μm filter fouled by beads*: (a) transmission and reflection images of a membrane, (b) a membrane fouled by 5.1μm beads, (c) a membrane fouled by 8.9μm beads.

Filtering chronic myeloid leukemia K562 cells

In Fig.S9a–f, the size distribution of K562 cells before filtering and after filtering using UTM-5~9μm are shown. In Fig.S9h, D50, D90, D95, and D99 before filtering and after filtering using UTM-5~9μm are shown. As the filter pore diameter becomes smaller, the size distribution after filtering tends to move away from the original solution. These results indicate that the developed filter can be used for the separation of soft spherical cells.

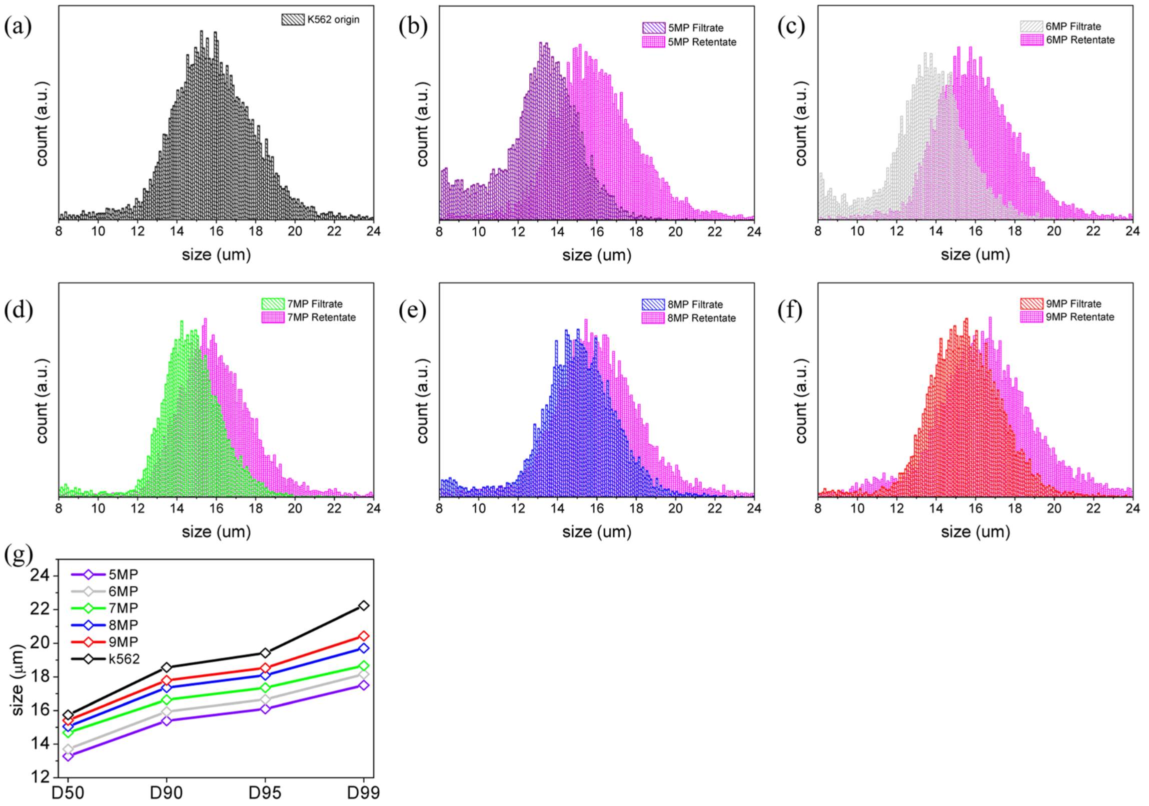

Figure S9. *Filtering result for K562 cells*: (a) the cell size distribution of K562 cells, (b) distributions of filtrate and retentate after filtration with UTM-5μm filter, (c) distributions after filtration with UTM-6μm, (d) distributions after filtration with UTM-7μm, (e) distributions after filtration with UTM-8μm, (f) distributions after filtration with UTM-9μm, (g) D50 to D99 of the original k562 cells and filtrates of 5-9μm filters.

Finite element analysis result about various filtering conditions

In the actual filtering experiment, filtering was performed under the condition of pushing the top of the cell, and finite element analysis was performed based on this condition. Additionally, an analysis was performed for the condition of suctioning at the bottom of the filter. In the upper part of Fig. S10a, the analysis results for cell deformation under the suctioning condition are shown. Under the suction condition, it can be seen that the overall deformation is rather concentrated in the lower part of the cell. On the other hand, under the pushing condition, it can be seen that the deformation is distributed to the whole part of the cell. In Fig. S10b, the penetrating length of the cell was shown as a function of the applied pressure. Comparing the condition of pushing and suctioning, there is no significant difference when the pressure is low and the pore diameter is large. However, when the pressure is high and the pore diameter is small, the suctioning condition obviously shows a larger penetrating length than the pushing condition. This indicates the possibility that the suctioning condition can be effective for filtering, which requires experimental verification, and can be a motivation for further research in the future.

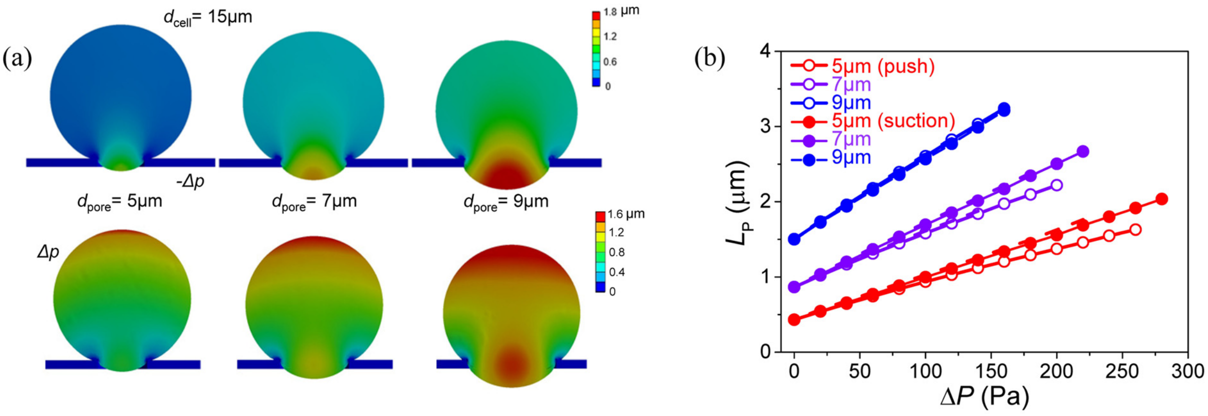

Figure S10. *Further finite element analysis (FEA) result about different filtering condition*: (a) deformation of cell entering the pores of different diameters, (the upper panel shows the condition of suctioning the cell by applying negative pressure from the bottom of the filter, and the lower panel shows the condition of pushing the cell by applying positive pressure from the top of the filter.) (b) FEA result of the penetrating length (L_P) as a function of the applied pressure (Δp). (two filtering conditions, 'push' and 'suction' were compared.)

References

46. Krizhevsky, A.; Sutskever, I.; Hinton, G.E. ImageNet Classification with Deep Convolutional Neural Networks. *Commun ACM* **2017**, 60, 84-90, doi:10.1145/3065386.

47. Lecun, Y.; Bottou, L.; Bengio, Y.; Haffner, P. Gradient-based learning applied to document recognition. *P IEEE* **1998**, 86, 2278-2324, doi:10.1109/5.726791.

48. ABAQUS Analysis User's Manual, Ver.6.6
